# Antiallergic and Antiarthritic Effects of Stem Bark Extract of *Glyphaea brevis* (Spreng) Monachino (Tiliaceae) in Murine Models

**DOI:** 10.1155/2013/874263

**Published:** 2013-09-17

**Authors:** David D. Obiri, Newman Osafo, Regina E. Abotsi

**Affiliations:** Department of Pharmacology, Faculty of Pharmacy and Pharmaceutical Sciences, College of Health Sciences, Kwame Nkrumah University of Science and Technology, Kumasi, Ghana

## Abstract

*Background*. Various parts of *Glyphaea brevis* (Spreng) Monachino (Tiliaceae) find a use in traditional medicine in the treatment of pain and oedema among others. This study evaluates the anti-inflammatory, antiallergic, and antiarthritic effects of a 70% (v/v) aqueous ethanol extract of the stem bark of *Glyphaea brevis* in murine models. *Materials and Methods*. The effect of the aqueous ethanol extract of *Glyphaea brevis* extract (GBE) was assessed on the maximal and total oedema responses in the carrageenan-induced paw oedema in mice to evaluate the acute anti-inflammatory actions of the extract. Systemic anaphylaxis was induced with compound 48/80 and survival rates monitored for 1 h in mice with prior treatment with GBE to assess the anti-allergic action of the extract. The indirect antihistamine effect of GBE was evaluated on clonidine-induced catalepsy. Rat adjuvant-induced arthritis model was used to study GBE's antiarthritic action. *Results*. GBE significantly suppressed the mean maximal swelling and the total paw swellings over 6 h in the carrageenan-induced paw oedema when administered either prophylactically or therapeutically. GBE dose dependently increased the time for compound 48/80-induced mortality. Administered either prophylactically or therapeutically, GBE inhibited clonidine-induced catalepsy while it had no effect on haloperidol-induced catalepsy. GBE caused a significant dose-dependent suppression of Freund's adjuvant-induced arthritis. *Conclusion*. *Glyphaea brevis* inhibits the *in vivo* degranulation of mast cells and thereby suppress allergy. In addition it exhibits anti-inflammatory action and attenuates Freund's adjuvant-induced arthritis. The results of this work contribute to validate the traditional use of *Glyphaea brevis* in the management of inflammatory disorders.

## 1. Introduction

Anaphylaxis is a rapid, life-threatening allergic reaction often triggered by food, drugs, insect venoms, latex, or allergen immunotherapy [1–5]. This acute allergic response is mediated by mast cells and basophils [6]. Mast cells express as one of their surface receptors the high affinity receptor for immunoglobulin E, IgE (Fc*ε*RI) [7] and play a central role in the inflammatory and immediate-type allergic responses that occur when sensitised individuals contact allergen through body surfaces [8]. In the absence of an antigen, the Fc*ε*RI binds to IgE. The major mechanism for the activation of these cells is the interaction of an antigen with the IgE-bound Fc*ε*RI. An alternative pathway of activation of the mast cell is offered by the so-called peptidergic pathway, a nonimmunologic mechanism. This is achieved by basic secretagogues of mast cells [9] which are polycationic and include compound 48/80. Stimulation of mast cells with IgE or compound 48/80 triggers the activation of signal transduction pathways which initiates a complex series of biochemical events leading to the release of mediators that cause allergic inflammation and anaphylactic reactions and delayed chronic inflammation [10–12]. One such chronic inflammatory disease is adjuvant-induced arthritis (AA) in rats which is characterized by infiltration of synovial membrane in association with destruction of joint and closely resembles rheumatoid arthritis in humans [13] as antibody to *Mycobacterial* antigen is reported in AA and also in rheumatoid arthritis.

The most commonly prescribed medication for the treatment of both acute and chronic inflammatory disorders such as allergy and rheumatoid arthritis, respectively, includes antihistamines, steroidal (glucocorticoids), nonsteroidal anti-inflammatory drugs (NSAIDs), disease-modifying antirheumatic (DMARDs) and immunosuppressant drugs. Primarily, though the goal of these drugs has been to relieve inflammation, prevent joint destruction, relieve pain, and to restore function of disabled joints, they are known to produce various side effects including gastrointestinal disorders, immunodeficiency, humoral disturbances, and even life-threatening effects [14–16]. In addition very recent findings have shown that glucocorticoids, the most potent anti-inflammatory drugs, are ineffective in anaphylaxis [17] or effective only when combined with a phosphatase inhibitor [18]. Consequently, the search for new potent regimens with less or no side effects especially from plant sources is heightened. In this regard, clinical benefits obtained with extracts from *Berberis*, *Tripterygium*, and *Urtica* species for treating rheumatic diseases are worth noting [19–21]. 

*Glyphaea brevis* is among the plants with potential anti-inflammatory actions used in traditional medicine in Ghana. Indeed, Dickson et al. have documented the anti-inflammatory action of *G. brevis* in the carrageenan-induced paw oedema in 7-day old cockerels [22]. *Glyphaea brevis* (Spreng) Monachino (family Tiliaceae) is a tree mainly present in forest regrowth, swampy places, rocky savanna, forest galleries, and fallow land. Different parts of the plant from the leaves through the stem bark to the roots have found a use in the traditional treatment of several disorders including chest pain and hepatitis and as an anticonvulsant [23–26]. Biological screen reveals that the leaves have antimicrobial [27] and antioxidant effects [28]. Phytochemical screening has shown the presence of cardiac glycosides, flavonoids, saponins, terpenoids, and tannins but no alkaloids [29]. Mbosso et al. identify linear long-chain aliphatic compounds, triterpenes, and steroids [30] as constituents.

In the current study, we demonstrate that the aqueous ethanol extract of *Glyphaea brevis* has inhibitory effects on the carrageenan-induced inflammation in mice. We subsequently show that *Glyphaea brevis* has suppressant effects on experimental immediate allergic reactions and also exhibits antiarthritic actions in mice and rats, respectively.

## 2. Materials and Methods

### 2.1. Collection of Plant Material

The stem bark of *Glyphaea brevis* was collected from the campus of the Forestry Research Institute of Ghana (FORIG) of the Council for Scientific and Industrial Research (CSIR), Fumesua, in January, 2012. The plant was authenticated by anatomical observation and direct comparison with the authentic specimens by A. Y. Yeboah-Mensah (Ph.D.) of the Department of Pharmacognosy, KNUST, and a specimen voucher GBE/T/010712 kept at the Department's herbarium.

### 2.2. Preparation of Plant Extract

The stem bark was air dried at room temperature chopped and milled into powder with a heavy duty blender (model 37BL85 (240CB6), Waring Commercial, USA). 2.0 kg of the powdered plant material was extracted by cold percolation with 4 L of 70% v/v ethanol for 5 days. The ethanol filtrate was concentrated under reduced pressure at 45°C by a vacuum rotary evaporator (model R-210, BUCHI, Switzerland) and further dried in an oven (Gallenkamp OMT, SANYO, Japan) to yield a solid mass of weight 107 g. The dried extract freshly dissolved in normal saline was referred to as GBE and was orally administered to experimental animals.

### 2.3. Animals

C57BL/6 and ICR mice (25–30 g) of both sexes and male Sprague-Dawley rats (180–200 g) were purchased from Noguchi Memorial Institute for Medical Research, University of Ghana, Accra, Ghana. The animals were kept in the Animal House of the Department of Pharmacology, College of Health Sciences, KNUST, Kumasi, Ghana, and allowed to acclimatize to the laboratory conditions (temperature 23 ± 2°C with a 12-hour light-dark cycle) for 14 days. Animals had free access to commercial pellet diet (GAFCO, Ghana) and water *ad libitum*. At the end of each experiment all animals used were euthanized ensuring that each animal was therefore used only once throughout the study. As per the internationally accepted principles for laboratory animal use and care (EEC Directive of 1986: 86/609 EEC), the animals were humanely handled throughout the experiment. Additionally all animal experiments were approved by the Department of Pharmacology, KNUST Ethics Committee (D/COL/GBE/2012 dated 24/02/2012).

### 2.4. Chemicals and Reagents

Compound 48/80 (C2313), *λ*-Carrageenan, and aspirin were purchased from Sigma-Aldrich (St Louis, USA). Clonidine was purchased from Boehringer Ingelheim Inc. (USA). Haloperidol was obtained from Janssen-Cilag Pty Ltd. (UK). Chlorpheniramine was supplied by DWD Pharmaceuticals Ltd. (India). Diclofenac and Liquid Paraffin were supplied by Troge (Germany) and Actavis Ltd. (UK), respectively.

### 2.5. Microorganism

Heat-killed *Mycobacterium tuberculosis* (strains C, DT, and PN (mixed)) was a kind donation from the Ministry of Agriculture, Fisheries and Food, UK.

### 2.6. Preparation of the Adjuvant

20 mg of heat-killed *Mycobacterium tuberculosis* (strains C, DT, and PN (mixed)) was finely ground in a mortar. Enough sterile liquid paraffin was added and triturated to 5 mg mL^−1^ suspension referred to as the Complete Freund Adjuvant, CFA. Sterile liquid paraffin only constitutes the Incomplete Freund Adjuvant, IFA.

### 2.7. Acute Anti-Inflammatory Study

#### 2.7.1. Induction of Carrageenan Paw Oedema

As earlier described by Winter et al., paw oedema was induced by injection of a 1% carrageenan suspension in normal saline (50 *μ*L, s.c.) into the subplantar tissue of the right hind paw of ICR mice (25–30 g) [31]. Oedema was monitored at 1 h intervals over 6 h with an electronic calliper (model Z22855, Milomex Ltd., Bedfordshire, UK) and expressed as the mean change in paw thickness. To determine the mean percent increase in paw thickness for each treatment, the following equation was used:

(1) $$\begin{array}{c} \%\;\;\text{increase}\;\;\text{in}\;\;\text{paw}\;\;\text{thickness}=\left[\frac{D_{t}-D_{0}}{D_{0}}\right]\times 100, \end{array}$$

where *D*_0_ and *D*_*t*_ are, respectively, the paw thickness before and at time *T* of the induction of the oedema.

Total oedema induced during the 6 h was determined as the area under the time course curves (AUC). To determine the percent inhibition of the total oedema for each treatment, the following equation was used:

(2) %  inhibition  of  oedema   =  [AUC(control)−AUC(drug  treatment)AUC(control)]×100.

Drug effects were evaluated by comparing the maximal and total oedema responses attained during 6 h in drug-treated groups with the corresponding values attained in saline-treated inflamed control groups. In the preventive (prophylactic) protocol, drug-vehicle, GBE 30, 100, and 300 mg kg^−1^, and aspirin 100 mg kg^−1^ was given orally 1 h prior to the induction of the oedema while, in the curative (therapeutic) protocol, treatment was done 1 h after oedema induction.

#### 2.7.2. Antianaphylactic Study

*Compound 48/80-Induced Systemic Anaphylaxis*. C57BL/6 mice (25–30 g) were given an i.p. injection of 10 mg kg^−1^ of the mast cell degranulator compound 48/80 to induce systemic anaphylaxis as previously described by Kim et al. [32]. Either drug-vehicle or GBE 30, 100, and 300 mg kg^−1^ was given orally 1 h prior to administration of compound 48/80. Mortality was monitored for 1 h after induction of anaphylaxis. 

### 2.8. Indirect Antihistamine Effect

#### 2.8.1. Induction of Clonidine Catalepsy

As earlier described by Ferre et al., clonidine (1 mg · kg^−1^, s.c.) was administered to 8–10-week-old ICR mice (20–25 g) in the bar test [33]. To test for catalepsy, rats were positioned so that their hindquarters were on the bench and their forelimbs rested on a 1 cm diameter horizontal bar, 10 cm above the bench. The time required to remove the paws from the bar was noted for each animal. The duration of catalepsy was measured at 30 min intervals up to 180 min after administration of clonidine. In the preventive (prophylactic) protocol, drug-vehicle (5 mL kg^−1^), GBE 30, 100, and 300 mg kg^−1^, and chlorpheniramine (10 mg kg^−1^) was given orally for 2 consecutive days ending 30 min before clonidine injection while, in the curative (therapeutic) protocol, treatment was done 60 min after catalepsy induction.

#### 2.8.2. Induction of Haloperidol Catalepsy

The same bar test, as earlier described by Ferre et al., was employed [33]. Haloperidol (1 mg · kg^−1^, s.c.) was administered to 8–10-week-old ICR mice (20–25 g). The duration of catalepsy was measured at 30 min intervals up to 180 min after administration of haloperidol. Drug-vehicle (5 mL kg^−1^), GBE (30, 100, and 300 mg kg^−1^), and chlorpheniramine (10 mg kg^−1^) were given orally for 2 consecutive days ending 30 min before haloperidol injection.

### 2.9. Chronic Anti-Inflammatory Study

#### 2.9.1. Induction of Rat Adjuvant Arthritis

Adjuvant arthritis was induced as previously described by Pearson. Briefly, the right hind paw of Sprague-Dawley rats (200–250 g) was injected intraplantar with 100 *μ*L of Complete Freund's Adjuvant (CFA) [34]. Arthritic control group received intraplantar injection of CFA, while nonarthritic control group received only intraplantar injection of 100 *μ*L sterile paraffin oil (Incomplete Freund's Adjuvant, IFA). Foot volume was measured by water displacement method with a plethysmometer (model 7140, Ugo Basile, Italy) as described by Fereidoni et al. [35] for the ipsilateral (injected) and contralateral (noninjected) hind limbs prior to intraplantar injection of CFA/IFA and daily for 28 days. The oedema component of inflammation was quantified by measuring the difference in foot volume between day zero and the various time points. Foot volumes were individually normalized as percentage of change from their values at day zero and then averaged for each treatment group. To determine the mean percent change in paw volume for each treatment, the following equation was used:

(3) $$\begin{array}{c} \%\;\;\text{change}\;\;\text{in}\;\;\text{paw}\;\;\text{volume}=\;\left[\frac{V_{t}-V_{0}}{V_{0}}\right]\times 100, \end{array}$$

where *V*_0_ and *V*_*t*_ are, respectively, the paw volumes before and at time *T* of the induction of the arthritis.

Total oedema induced during the acute phase (day 0–10) and polyarthritic phase (day 0–28) was determined as area under the time course curves (AUC). To determine the percent inhibition of the total oedema for each treatment, the following equation was used:

(4) %  inhibition  of  oedema   =[AUC(control)−AUC(drug  treatment)AUC(control)]×100.

Drug effects were evaluated by comparing the maximal and total oedema responses attained during 28 days in drug-treated groups with the corresponding values attained in saline-treated inflamed control groups. In the preventive (prophylactic) protocol, drug-vehicle, GBE 30, 100, and 300 mg kg^−1^, and diclofenac 6 mg kg^−1^, was given orally 1 h before the induction of the oedema on day zero and daily for 28 days while, in the curative (therapeutic) protocol, treatment was commenced on day 10 after oedema induction till day 28. All drugs were freshly prepared on each day of drug administration.

### 2.10. Statistical Analysis

All data were reported as mean values ± standard error of mean (s.e.m), *n* = 5. Time-course curves where appropriate were subjected to two-way (treatment × time) repetitive measures analysis of variance (ANOVA) with Bonferroni's post hoc test. Differences in AUCs were analysed by a one-way ANOVA followed by a Student-Neuman-Keul's range test. In case of the lethality rate, the Kaplan-Meier Survival plots were used. Differences were considered significant at *P* ≤ 0.05. All graphs were plotted with GraphPad prism for Windows Version 5.00 (GraphPad, San Diego, CA, USA). 

## 3. Results

### 3.1. Effect of *G. brevis* Extract (GBE) on Carrageenan-Induced Paw Oedema

**Figure 1:**
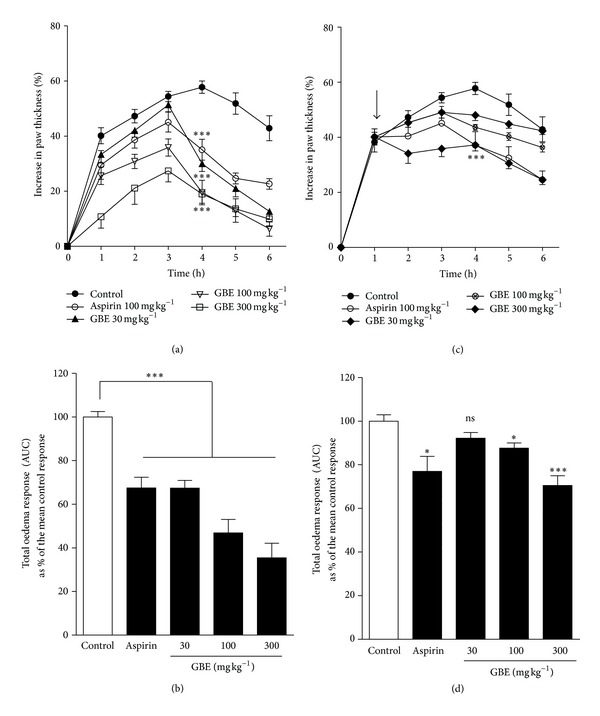
Effect of *G. brevis* extract (GBE) on carrageenan-induced oedema in mice. ICR mice (25–30 g) were injected with 50 *μ*L of a 1% carrageenan suspension into the subplantar tissue of the right hind paw. Oedema was monitored at 1 h intervals over 6 h as percentage increase in paw thickness (a and c), and total oedema induced during the 6 h was calculated as area under the time course curves, AUC (b and d). Drug effects were evaluated by comparing the maximal and total oedema responses attained during 6 h in drug-treated groups with the corresponding values attained in drug vehicle-treated inflamed control groups. In the preventive (prophylactic) protocol (left panel), drug-vehicle, GBE 30, 100, and 300 mg kg^−1^, and aspirin 100 mg kg^−1^ were given orally 1 h before the induction of the oedema while, in the curative (therapeutic) protocol (right panel), treatment was done 1 h after oedema induction. Data is presented as mean ± s.e.m. (*n* = 5). ***P ≤ 0.0001, **P ≤ 0.01, and **P ≤ 0.05 when compared with control. Arrow indicates point of extract administration in the therapeutic protocol.

As carrageenan-induced oedema culminates in degranulation involving the release of histamine and other autocoids from allergen-specific IgE-activated mast cells which mediate the initial phase of the acute inflammatory response, we examined the effect of GBE on acute inflammation in the mouse. To investigate the anti-inflammatory effect of GBE, we injected 50 *μ*L of a 1% carrageenan suspension into subplantar tissue of the right hind paw of mice. Drug effects were evaluated by comparing the maximal and total oedema responses attained during 6 h in drug-treated groups with the corresponding values attained in the saline-treated control groups before and after the induction of the oedema. When administered before (preventive) the induction of the carrageenan paw oedema, GBE (30, 100, and 300 mg kg^−1^) dose dependently caused the mean maximal swelling attained during 6 h to be significantly (*P* ≤ 0.0001) reduced, respectively, to 30.00 ± 2.84%, 19.56 ± 4.43%, and 19.02 ± 3.36% of the mean inflamed control response of 57.76 ± 2.22% (Figure 1(a)). The total paw swellings induced over the 6 h (measured as the area under the time course curve, AUC) were also dose dependently and significantly (*P* ≤ 0.0001) suppressed, respectively, to 67.40 ± 3.55%, 46.89 ± 6.15%, and 35.45 ± 6.70% of the inflamed control response (Figure 1(b)) corresponding, respectively, to 32.60 ± 4.33%, 53.11 ± 6.63%, and 64.55 ± 7.15% inhibitions of the mean total oedema response. *G. brevis* administered in the same doses after the induction (curative) of the carrageenan paw oedema significantly (*P* ≤ 0.0002) suppressed the mean maximal swelling attained during the 6 h, respectively, to 48.06 ± 1.96%, 43.62 ± 1.10%, and 37.24 ± 2.18% of the mean inflamed control response of 57.76 ± 2.22% (Figure 1(c)). However, the total paw swellings induced over the 6 h were significantly suppressed, respectively, to 87.71 ± 2.23% and 70.55 ± 4.43% of the mean control response at 100 and 300 mg kg^−1^ (Figure 1(d)) corresponding, respectively, to 12.29 ± 3.73% and 29.45 ± 5.31% inhibitions of the total oedema response. Aspirin (100 mg kg^−1^), a positive control drug, significantly suppressed all the parameters under study (Figures 1(a)–1(d)).

### 3.2. Effect of *G. brevis* Extract (GBE) on Compound 48/80-Induced Systemic Anaphylaxis

**Figure 2:**
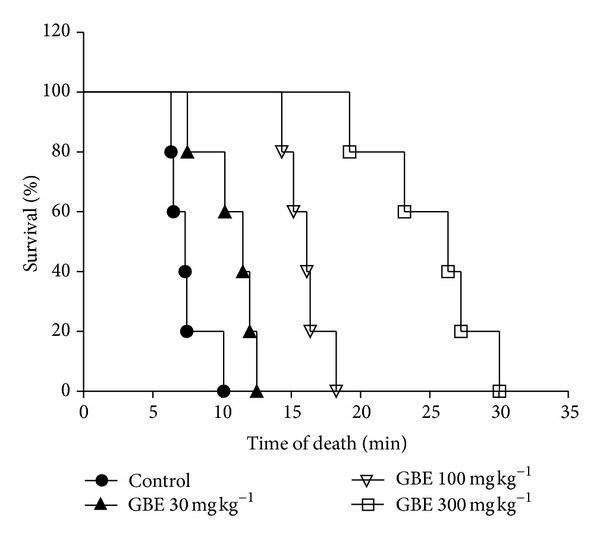
Effect of *G. brevis* extract (GBE) on compound 48/80-induced anaphylaxis in mice. C57BL/6 mice (25–30 g) were pretreated for 1 h with saline, GBE 30, 100, and 300 mg kg^−1^ (*n* = 5). Compound 48/80 was injected (10 mg kg^−1^, i.p.) and mortality monitored for 1 h after induction of anaphylactic shock. The experimental results were analysed using the Log-rank (Mantel Cox) test and the survival rates were significant ***P ≤ 0.0002 compared to the saline-treated control mice.

As allergen-induced degranulation of mast cell is a component of allergy and an extreme form of the allergic reaction is anaphylaxis, we investigated whether GBE exerts an inhibitory effect on anaphylaxis. To determine the effect of GBE on this allergic reaction, an *in vivo* model of a systemic reaction was used. Compound 48/80 (10 mg kg^−1^) was used as a model for induction of a systemic fatal allergic reaction. Injection of compound 48/80 into mice induced fatal shock in 100% of animals in 10 min. When administered (30, 100, and 300 mg kg^−1^) GBE caused the latent time for the death of mice injected with compound 48/80 to be significantly and dose dependently increased to 12.5, 18.25, and 30 min, respectively (Figure 2).

### 3.3. Effect of *G. brevis* Extract (GBE) on Clonidine-Induced Catalepsy

**Figure 3:**
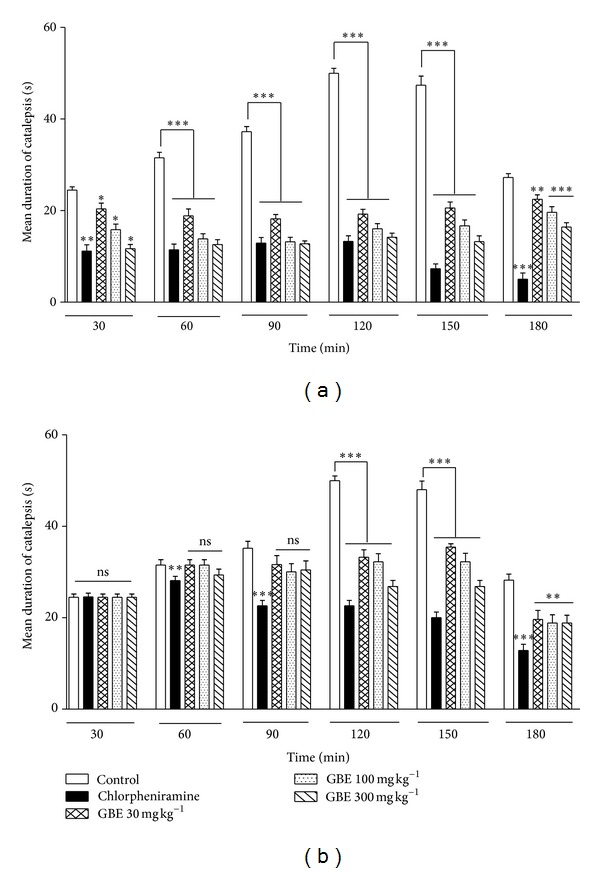
Effect of *G. brevis* extract (GBE) on clonidine-induced catalepsy. 8–10-week-old ICR mice (20–30 g) received clonidine 1 mg · kg^−1^, s.c. and their forepaws placed on a horizontal bar (1 cm in diameter, 10 cm above the table). The duration of catalepsy was measured at 30 min intervals up to 180 min after administration of clonidine. (a) In the preventive (prophylactic) protocol, vehicle (5 mL kg^−1^), GBE (30, 100, and 300 mg kg^−1^), and chlorpheniramine (10 mg kg^−1^) were given orally for 2 consecutive days ending 30 min before clonidine injection. (b) In the curative (therapeutic) protocol drug treatment commenced 1 h after induction of catalepsy. Values are mean ± s.e.m. (*n* = 5). Data was subjected to two-way (treatment × time) repetitive measures analysis of variance (ANOVA) with Bonferroni's post hoc test. Significance between groups of vehicle and drug/extract-treated mice ***P ≤ 0.0001, **P ≤ 0.01, *P ≤ 0.05, ns is nonsignificant.

As histamine is frequently used as an indicator of mediator release in IgE-dependent immediate-type anaphylaxis, we studied the indirect antihistamine effect of the extract on histamine release from mast cells in clonidine-induced catalepsy. Catalepsy was induced in all the mice after the administration of clonidine (1 mg kg^−1^, s.c) and this peaked at 120 min in the vehicle-treated control group. GBE (30, 100, and 300 mg kg^−1^) and chlorpheniramine (10 mg kg^−1^) showed significant dose-dependent inhibition of clonidine-induced catalepsy at all time points in the preventive protocol (Figure 3(a)). In the curative protocol, both chlorpheniramine and GBE significantly suppressed the clonidine-induced catalepsy from 120 min to 180 min (Figure 3(b)).

### 3.4. Effect of *G. brevis* Extract (GBE) on Haloperidol-Induced Catalepsy

**Figure 4:**
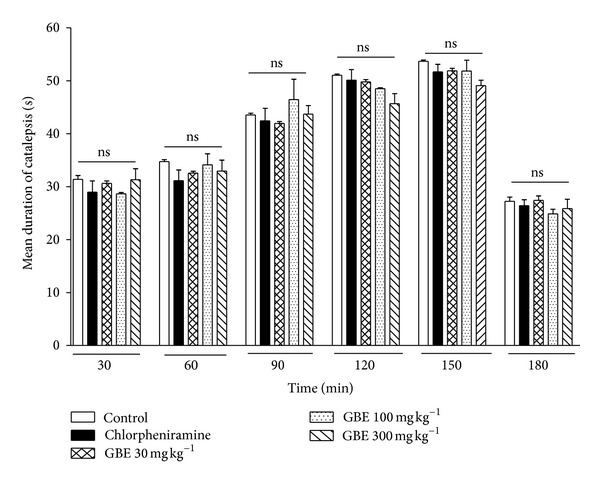
Effect of *G. brevis* extract (GBE) on haloperidol-induced catalepsy. 8–10-week-old ICR mice (20–30 g) received haloperidol 1 mg · kg^−1^, s.c. and their forepaws placed on a horizontal bar (1 cm in diameter, 10 cm above the table). The duration of catalepsy was measured at 30 min intervals up to 180 min after administration of haloperidol. Drug-vehicle (5 mL kg^−1^), GBE (30, 100, and 300 mg kg^−1^), and chlorpheniramine (10 mg kg^−1^) were given orally for 2 consecutive days ending 30 min before haloperidol injection. Values are mean ± s.e.m. (*n* = 5). Data was subjected to two-way (treatment × time) repetitive measures analysis of variance (ANOVA) with Bonferroni's post hoc test. Differences were considered significant at *P* ≤ 0.05, ns is nonsignificant.

As catalepsy can also be induced by neuroleptic drugs and is a common extrapyramidal side effect of drugs that either increase histamine release in the brain or inhibit dopaminergic transmission, we studied the effect of *G. brevis* on haloperidol-induced catalepsy in mice. Catalepsy was observed in all the groups peaking at 150 min in the vehicle control group after the administration of haloperidol (1 mg kg^−1^, s.c). GBE (30, 100, and 300 mg kg^−1^) and chlorpheniramine (10 mg kg^−1^) showed no significant inhibition of haloperidol-induced catalepsy at all time points studied (Figure 4).

### 3.5. Effect of *G. brevis* Extract (GBE) on Rat Adjuvant-Induced Arthritis

**Figure 5:**
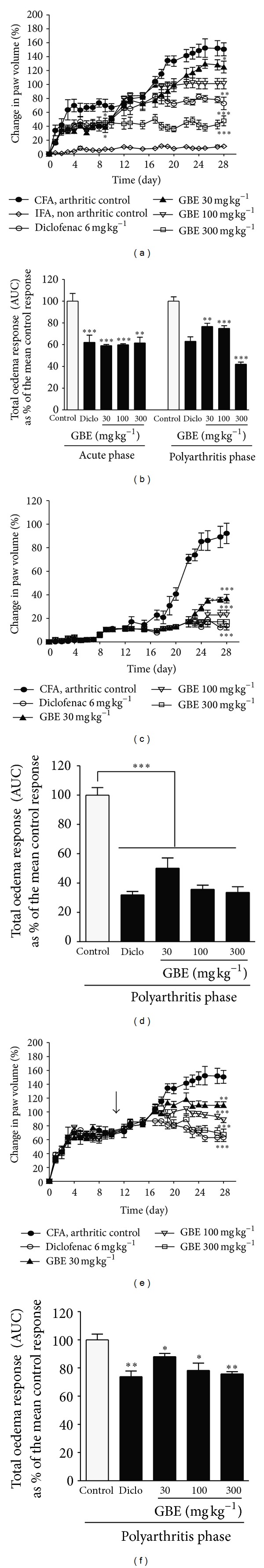
Effect of *G. brevis* extract (GBE) on adjuvant-induced arthritis. Sprague-Dawley rats (200–250 g) were injected intraplantar with 100 *μ*L of CFA or IFA into the right hind paw. Foot volume was measured by water displacement plethysmography daily for 28 days. The oedema component of inflammation was monitored as the percentage change in paw volume (a, c, and e) and the total oedema induced during the acute and polyarthritis phases calculated as area under the time course curves, AUC (b, d, and f). In the preventive protocol (top and middle panels), drug vehicle, diclofenac 6 mg kg^−1^, or GBE 30, 100, and 300 mg kg^−1^ were given orally 1 h before the induction of the arthritis and daily for 28 days while, in the curative protocol (bottom panel), treatment commenced 10 days after arthritis induction. Drug effects were evaluated by comparing the maximal and total oedema responses attained during 28 days in drug-treated groups with the corresponding values attained in drug-vehicle-treated inflamed control groups. Data is presented as mean ± s.e.m. (*n* = 5) ***P ≤ 0.0001, **P ≤ 0.01, **P ≤ 0.05 when compared with vehicle-treated control group. Arrow indicates point of extract administration in the therapeutic protocol.

Since events of both carrageenan-induced oedema and the primary phase of adjuvant-induced arthritis correspond to those in the early exudative phase of inflammation which is an important feature of inflammatory pathology, we studied the effect of the extract on rat adjuvant-induced arthritis, a model of chronic inflammation. Adjuvant arthritis was induced in the right hind paw of rats with an intraplantar injection of CFA. Drug effects were evaluated by comparing the maximal and total oedema responses attained during 28 days in drug-treated groups with the corresponding values attained in control groups before and after the induction of the oedema on both the ipsilateral (injected) and contralateral (noninjected) limbs. All arthritic control rats showed acute inflammatory oedema at the ipsilateral paw (Figures 5(a) and 5(e)) followed by subsequent chronic polyarthritic phase in which the inflammation had spread to the contralateral limb (Figure 5(c)). There was no significant change throughout the study in the paw volume of the noninflamed control groups that were injected with IFA (Figure 5(a)). 

Daily prophylactic administration over 28 days of diclofenac 6 mg kg^−1^ and GBE at 30, 100, and 300 mg kg^−1^ produced a significant (*P* ≤ 0.001) reduction of the maximal adjuvant-induced swelling to 72.80 ± 8.96%, 125.08 ± 8.49%, 101.25 ± 7.43%, and 47.75 ± 7.12%, respectively, compared to the mean maximal control swelling of 150.58 ± 9.34% (Figure 5(a)). The total adjuvant-induced response (AUC) over 28 days (polyarthritis) was dose dependently significantly (*P* ≤ 0.0001) reduced to 63.14 ± 4.09%, 76.77 ± 2.93%, 75.05 ± 2.41%, and 42.07 ± 1.92% (Figure 5(b)), respectively, of the mean inflamed group response corresponding, respectively, to 36.86 ± 5.77%, 23.23 ± 5.02%, 24.95 ± 4.73%, and 57.93 ± 4.50% inhibitions of the total oedema response in the ipsilateral limb. On the acute phase of the arthritic response (day 0–10), the mean maximal oedema response was significantly (*P* ≤ 0.05) reduced to 50.34 ± 4.61%, 51.80 ± 1.75%, 51.08 ± 1.40%, and 44.87 ± 7.41%, respectively, compared to the mean maximal control response of 66.95 ± 5.42% (Figure 5(a)) while the total adjuvant-induced response (AUC) over 10 days was significantly (*P* ≤ 0.002) reduced to 62.19 ± 6.62%, 59.08 ± 1.06%, 59.99 ± 0.97%, and 61.59 ± 5.2% (Figure 5(b)) presenting 37.81 ± 9.67%, 40.92 ± 7.12%, 40.01 ± 7.11%, and 38.41 ± 8.78% inhibitions of the total oedema response, respectively.

On the contralateral hind limb, diclofenac and GBE administered prophylactically significantly (*P* ≤ 0.0001) suppressed the mean maximal oedema swelling in 28 days to 13.85 ± 3.61%, 30.76 ± 3.66%, 22.87 ± 4.15%, and 17.12 ± 4.35% relative to the mean maximal control swelling of 92.20 ± 8.95% (Figure 5(c)) while the total oedema response was in a dose-dependent manner significantly (*P* ≤ 0.001) reduced to 32.00 ± 2.29%, 50.14 ± 6.98%, 35.73 ± 2.76%, and 33.62 ± 3.94%, respectively, of the mean control response (Figure 5(d)) corresponding to 68.00 ± 5.62%, 49.86 ± 8.66%, 64.27 ± 5.83%, and 66.38 ± 6.47% inhibitions of the total oedema response, respectively.

In a separate experiment where drug administration commenced 10 days after the induction of the arthritis (curative), daily administration of diclofenac 6 mg kg^−1^ and the *Glyphaea brevis* extract 30, 100, and 300 mg kg^−1^ on the ipsilateral limb caused a significant (*P* ≤ 0.001) suppression of the mean maximal adjuvant-induced swelling on the 28th day, respectively, to 63.10 ± 5.91%, 109.60 ± 5.03%, 88.41 ± 3.79%, and 67.42 ± 7.91% of the mean maximal control swelling of 150.58 ± 9.34% (Figure 5(e)). The total adjuvant-induced swelling (AUC) during the experimental period was also dose dependently significantly (*P* ≤ 0.05) suppressed, respectively, to 78.83 ± 3.92%, 88.03 ± 2.32%, 78.31 ± 5.09%, and 75.89 ± 1.50% (Figure 5(f)) presenting 26.17 ± 5.66%, 11.97 ± 4.69%, 21.69 ± 6.52%, and 24.11 ± 4.34% inhibitions of the total oedema response, respectively. On the contralateral limbs GBE effects were similar as described (results not shown). 

## 4. Discussion

In this report, we show that oral treatment of rodents with 70% aqueous ethanol extract of* Glyphaea brevis* exerts inhibitory effects on carrageenan-induced oedema, a model of acute inflammation, systemic anaphylaxis, and adjuvant-induced arthritis, a model of chronic inflammation.

Carrageenan-induced mouse paw oedema has a wide application in the test of new anti-inflammatory drugs as well as in the study of mechanisms involved in inflammation [36] and is reported to exhibit a biphasic response with the first phase mediated by histamine and serotonin while the second phase is by prostaglandins with nitric oxide with kinins maintaining the oedema between the two phases [37]. A dose-dependent suppression of the oedema was displayed by the extract when it was given prophylactically. Our findings in the mouse agrees with an earlier report on the inhibitory effect of the aqueous ethanol extract of the stem bark of *Glyphaea brevis* on acute inflammation in the carrageenan-induced oedema model in 7-day-old cockerels [22]. The extract administered after the onset of the inflammatory reaction could also suppress significantly both the maximal oedema response and the total oedema response. The ability of the extract to exert anti-inflammatory effects both prophylactically and therapeutically suggests that it possibly acts by inhibiting the release, synthesis, and/or action of mediators involved in acute inflammation. The exact mechanism, however, needs to be established.

Anaphylactic shock is an extreme and fatal allergic reaction, and in mice it is reportedly mediated by two pathways: an IgE-dependent pathway with IgE-mast cells as the major player [38] and another pathway mediated by IgG1 with macrophages and basophils as the major players [39]. The early clinical and experimental evidence suggests that systemic anaphylaxis mediated by mast cell degranulation results in the rapid release of preformed mediators, including histamine, proteoglycans, serotonin, lipid-derived mediators, and cytokines [12, 40]. These mediators reportedly act on target cells to present with cardiovascular effects of vasodilation, increased vascular permeability, hypotension, bronchospasm and, as a result, shock [41]. The main organs involved in the early stages of anaphylaxis are the skin (urticaria and angioedema) and the respiratory tract (laryngeal oedema and bronchospasm). However, dysfunction of the central and peripheral cardiovascular systems usually dictates the outcome of anaphylactic events [42]. A drug capable of suppressing or preventing these effects must exhibit a potent antiallergic action. Experimentally, compound 48/80 stimulates mast cells and initiates the activation of signal transduction pathways, through the activation of G proteins [43, 44] leading to the release of the proinflammatory mediators such as histamine, heparin, lipid-derived mediators, and various cytokines [12]. Eventually, a perturbation in the membrane [45] occurs as these mediators cause an increase in the permeability of the lipid bilayer membrane. Our findings showing that prior treatment of the mice with GBE was able to exert a delay in the time of mortality of the animals that were injected with the compound 48/80 suggest that GBE possibly stabilizes the lipid bilayer membrane. This in turn significantly suppressed the perturbation from being induced by compound 48/80 and therefore regulated the *in vivo* degranulation of the mast cells in the mice through stabilizing the membrane fluidity. In addition to inhibiting the degranulation of mast cells, GBE exhibits anti-inflammatory activity which might act in synergy with its antiallergic effect in the treatment of allergic inflammation.

Catalepsy, a condition in which an animal maintains an imposed posture for a long time before regaining normal posture, is a sign of extrapyramidal effect of drugs that increase histamine release in the brain or inhibit dopaminergic transmission. Histamine is frequently used as an indicator of mediators in IgE-dependent immediate-type anaphylaxis. While Schwartz reports that histamine containing mast cells have been identified in the brain [46], Chopra and Dandiya also demonstrate that different stages of catalepsy directly correlate with the histamine content of the brain [47]. It is also established that while clonidine releases histamine from mast cells in a similar manner to a selective degranulator compound 48/80 [48], it causes degranulation without causing any damage to the cell wall [49]. Jadhav et al. observed that clonidine-induced catalepsy in the mouse is mediated by histamine release from the mast cells acting via H_1_ receptors [50] and consequently inhibited by histamine H_1_ receptor antagonists but not by H_2_ receptor antagonists [51]. In this study, our results revealed a dose-dependent suppression of clonidine-induced catalepsy by both GBE and the H_1_-receptor antagonist, chlorpheniramine, when administered either prophylactically or therapeutically and is strongly indicative of an indirect antihistaminic H_1_ receptor activity of the extract. Haloperidol, a nonselective D2 dopamine antagonist, also induces catalepsy by primarily blocking the dopamine receptors in the striatum and agents that increase dopaminergic transmission inhibit haloperidol-induced catalepsy [52]. In our study, both the antihistaminic drug chlorpheniramine and GBE failed to inhibit haloperidol-induced catalepsy consistent with the findings that haloperidol-induced catalepsy is independent of histamine release. Again failure of the extract to inhibit haloperidol-induced catalepsy also demonstrates that GBE may not have any effect on dopaminergic transmission. Taken together with the effect of the extract on compound 48/80-induced anaphylaxis, GBE possibly possesses antihistaminic activity and mast cell stabilizing ability in a dose-dependent manner and consistent with earlier findings that extracts with antihistaminic or mast cell stabilizing effect inhibit clonidine-induced catalepsy [51] and as reported for *Allium sativum* and *Terminalia belerica*, *Clerodendrum serratum*, and *Ficus benghalensis* [53–55].

As events in carrageenan-induced oedema are very similar to those occurring during the primary phase of adjuvant-induced arthritis and these correspond to those in the early exudative phase of inflammation [56, 57], we investigated the effect of GBE on Freund's rat adjuvant-induced arthritis. Pearson and Wood report that rat adjuvant arthritis (AA) is a chronic, polyarticular, erosive type of arthritis induced by an injection of heat-killed mycobacteria [58]. AA is widely used for studying the pathogenesis of rheumatoid arthritis, RA, and for searching new drugs for treatment of rheumatoid disease [59–61]. In the present study, rats were selected to induce arthritis because rats develop a chronic swelling in multiple joints with influence of inflammatory cells, erosion of joint cartilage, and bone destruction. These inflammatory changes ultimately result in the complete destruction of joint integrity and functions in the affected rats [62] through the release of number of mediators like cytokines such as interleukin-1B, (IL-IB), tumor necrosis factor alpha (TNF-*α*), Granulocyte macrophage colony-stimulating factor (GM-CSF), interferon's, and prostaglandins such as (PGDF) [61, 63]. Turull and Queralt showed that in the pathogenesis of adjuvant arthritis in the rat autoantigens that cross-react with *Mycobacteria* are implicated and that adjuvant arthritis appears as a consequence of an immune response to cell wall of *Mycobacterium*. Compared with normal rats, AA rats had higher levels of IgG anti-*Mycobacterium* antibodies and the delayed skin reactions induced by the soluble fraction of *Mycobacterium* [64]. GBE at doses used significantly decreased humoral immune responses. At the same time, treatment with GBE at all three doses assayed inhibited the delayed-type hypersensitivity seen in arthritic animals. This work suggests that GBE might exert its effect through its influence on the cellular and on the humoral immune response to the *Mycobacterium* in adjuvant-induced arthritic rats. Hoffmann et al. indicate that prognosis of rat adjuvant-induced arthritis can be divided into three phases as in human rheumatoid arthritis. These phases start with the induction phase which has no evidence of synovitis, followed by early synovitis, and finally late synovitis with progressive joint destruction [65]. A good antirheumatic agent should be able to block one or more of these phases. The aqueous ethanol extract, GBE, used in the work suppressed joint inflammation and synovitis. GBE suppressed the swelling associated with both the acute and polyarthritic phases of adjuvant-induced arthritis when used both prophylactically and therapeutically. This is significant because Kaibara et al. could demonstrate that cyclosporine, an immunosuppressive drug, prevented the onset of collagen-induced arthritis in rats; however, when used after the disease had been established, it exacerbated the condition [66]. Again as shown by Larsson et al. limonide, an experimental drug developed against heterologous collagen-induced arthritis, exhibited similar paradoxical effects [67]. These earlier findings strongly suggest that demonstrated anti-inflammatory effect of a drug given prior to the induction of inflammation does not necessarily guarantee the same effect when given after the induction of the inflammation. Therefore on both the carrageenan-induced paw oedema and rat adjuvant-induced arthritis models, the ability of GBE to exert anti-inflammatory effects both prophylactically and therapeutically support the presence of compounds most likely exerting inhibitory effects through interference with the pathophysiological processes underlying inflammation. The exact mechanism, however, needs to be established. In addition to reducing the general swelling, the extract prevented the systemic spread of the arthritis to the noninjected limb suggesting an effective anti-inflammatory action.

Interestingly, phytochemical screening of the plant revealed the presence of several therapeutically valued constituents including flavonoids, tannins [26, 28], and steroids [30]. Therefore several mechanisms could be responsible for the actions of the extract. For example, flavonoids have powerful antioxidant actions [68, 69] and exhibit significant analgesic activity primarily by targeting and inhibiting prostaglandins, PGs [70, 71], through inhibition of eicosanoid biosynthesis. PGs are the end products of the *cyclooxygenase* and *lipoxygenase* pathways [72] and are implicated in various immunological responses. Again flavonoids suppress the intracellular Ca^2+^ ion elevation and consequently depress the release of proinflammatory mediators such as TNF*α* [73]. In this regard, flavonoids such as quercetin have potent effects on acute inflammation [74]. Tannins are also known to be potent inhibitors of *cyclooxygenase*-1 (COX-1) and with antiphlogistic activity [75]. The mechanisms of anti-inflammatory activity may be in part accounted for by the antiphlogistic action of the tannins. Steroids (glucocorticoids) as anti-inflammatory agents act by different mechanisms via the steroid receptor to regulate gene transcription either positively (transactivation) or negatively (transrepression) [76, 77]. Again, cross-talk between the steroid and the signalling pathways which are triggered on mast cell activation serves as another mode of their action since some upstream molecules are important in the phosphorylation of transcription factors [78]. In sum, the anti-inflammatory actions of the total crude extract are attributed to effects of the individual constituents. The established anti-inflammatory properties of *G. brevis*, warrant further evaluation of its clinical utility in mast cell-mediated immediate and delayed allergic diseases.

## 5. Conclusion

The extract of *Glyphaea brevis* inhibits *in vivo* degranulation of mast cells and thereby suppresses allergy through inhibition of histamine release from mast cells. Additionally, *Glyphaea brevis* exhibits anti-inflammatory activity and attenuates Freund's adjuvant-induced arthritis.
